# Evolution and Trade-Off Dynamics of Functional Load

**DOI:** 10.3390/e24040507

**Published:** 2022-04-05

**Authors:** Erich Round, Rikker Dockum, Robin J. Ryder

**Affiliations:** 1 Surrey Morphology Group, University of Surrey, Guildford GU2 7XH, UK; 2 Ancient Language Lab, University of Queensland, St Lucia 4072, Australia; 3 Max Planck Institute for the Science of Human History, D-07745 Jena, Germany; 4 Department of Linguistics, Swarthmore College, Swarthmore, PA 19081, USA; rdockum1@swarthmore.edu; 5 CEREMADE, CNRS, UMR 7534, Université Paris-Dauphine, PSL University, 75016 Paris, France; ryder@ceremade.dauphine.fr

**Keywords:** functional load, phonology, phylogenetic signal, phylogenetic correlation, homophony avoidance, sound change, Pama–Nyungan, transphonologization, parallel evolution, Sapir’s drift

## Abstract

Functional load (FL) quantifies the contributions by phonological contrasts to distinctions made across the lexicon. Previous research has linked particularly low values of FL to sound change. Here, we broaden the scope of enquiry into FL to its evolution at higher values also. We apply phylogenetic methods to examine the diachronic evolution of FL across 90 languages of the Pama–Nyungan (PN) family of Australia. We find a high degree of phylogenetic signal in FL, indicating that FL values covary closely with genealogical structure across the family. Though phylogenetic signals have been reported for phonological structures, such as phonotactics, their detection in measures of phonological function is novel. We also find a significant, negative correlation between the FL of vowel length and of the following consonant—that is, a time-depth historical trade-off dynamic, which we relate to known allophony in modern PN languages and compensatory sound changes in their past. The findings reveal a historical dynamic, similar to transphonologization, which we characterize as a flow of contrastiveness between subsystems of the phonology. Recurring across a language family that spans a whole continent and many millennia of time depth, our findings provide one of the most compelling examples yet of Sapir’s ‘drift’ hypothesis of non-accidental parallel development in historically related languages.

## 1. Introduction

Functional load (FL) quantifies the contribution of specific phonological contrasts to distinctions made in the lexicon of a language [1,2,3]. In English for example, the phonemes /t/ and /d/ contrast, and thus there exist phonological strings in English, including consonant clusters, syllables, and whole words, which differ only by virtue of one containing /t/ in a position where the other contains /d/. Examples within word-length strings include *time*/*dime*, *welter*/*welder*, and *hit/hid*. At a conceptual level, the FL of the {/t/,/d/} contrast in English is the degree to which that contrast supports the distinctiveness of phonological strings in the English lexicon, or conversely, the degree to which those strings would be conflated if /t/ and /d/ were merged into a single category.

A classic operationalization of FL by Hockett [2] is in terms of entropy [4]. Hockett’s definition makes reference to domains, D, which could be words, morphemes, syllables, or any kind of substring composed of phonemes. In a language L, the lexicon Λ will contain a set $S_{D,\Lambda }$ of unique phonological string types, *s*, which comprise a domain of type D. The entropy of domain D in lexicon Λ is:

(1) $$H_{D,\Lambda }=-\sum_{s\in S_{D,\Lambda }}\mathrm{Pr}\left(s\right)\cdot \log_{2}\mathrm{Pr}\left(s\right)$$

Following Hockett [2], the FL of a phonological contrast ϕ in lexicon Λ and domain D is the difference between two entropy measures: the entropy of domain D in lexicon Λ, and of domain D in an altered lexicon $\Lambda_{\phi }^{\prime }$, created by collapsing the contrast ϕ in Λ:

(2) $$f\left(D,\Lambda ,\phi \right)=H_{D,\Lambda }-H_{D,\Lambda_{\phi }^{\prime }}$$

A related metric is a normalized functional load, which states the FL of a contrast relative to the entropy of domain D in lexicon Λ [3]:

(3) $$f_{norm}\left(D,\Lambda ,\phi \right)=\frac{H_{D,\Lambda }-H_{D,\Lambda_{\phi }^{\prime }}}{H_{D,\Lambda }}$$

A phonological contrast, ϕ, may refer to distinctions between the members of a single set of phonemes, such as between two phonemes {/t/, /d/} or between four phonemes {/t/, /d/, /s/, /z/}. Alternatively, it may refer to a collection of multiple, parallel distinctions, defined by a collection of sets and the distinctions within each of them. For instance, a contrast between voiced and voiceless stop consonants could refer to the distinctions within each of the three sets in the collection {{/p/, /b/}, {/t/, /d/}, {/k/, /g/}}, and a contrast between the places of articulation of stop consonants could refer to the distinctions within each of the two sets in the collection {{/p/, /t/, /k/}, {/b/, /d/, /g/}}. In all cases, the altered lexicon $\Lambda_{\phi }^{\prime }$ is obtained by taking each individual set and replacing the phonemes within it with a single phonemic symbol that is distinct from all other phonemic symbols in L.

As FL is a frequency measure calculated from empirical datasets, the results that one obtains will vary according to the corpus used [3,5]. Accordingly, when investigating FL, it is important to bear in mind how the properties of a dataset might relate to the research question and to any assumptions relevant to the interpretation of results.

### 1.1. Functional Load and Explanation in Linguistics

Human linguistic communication is a highly complex cultural and cognitive phenomenon. One of its key traits is that every language contains an inventory of thousands of distinctive symbolic units termed *morphemes*, which in turn, comprise smaller, distinctive phonological elements [6]. In spoken languages, these include *phonemes* (found in all languages) and lexically distinctive prosodic elements, such as tones, accent and stress patterns (found in around half of all languages [7,8,9]). Of equal significance is that, although the existence of morphemes and phonemes is universal, the actual inventories are language-specific. In linguistics, this specificity of individual languages gives rise to fields of enquiry, such as linguistic typology, studying the range and distribution of variation across languages, and historical linguistics, studying the evolution of distributions over time. Both subfields contribute to the scientific understanding of human language in general by generalizing across the variation of individual language systems.

To better understand how linguistic systems provide and organize the distinctiveness that makes linguistic communication possible, investigations of FL play a valuable role in shedding light on how distinctions in phonological modules function to support distinctiveness in larger strings. Currently, in both typology and historical linguistics, the study of FL is in its infancy with much of the potential scope of FL research still to be explored. Most studies of FL, for instance, have focused on words as the strings of interest (see Section 1.2 below). However, words are only one kind of string known to be significant in the structure and use of linguistic systems. Systems of phonological structure exhibit well-known organizational principles at levels above the phoneme and below the word [10,11], while psycholinguistic research shows that listeners also use multi-phonemic strings smaller than the word or morpheme, to gain efficiencies in speech processing [12]. The study we present here illustrates some of the potential of studying strings other than words.

FL research to date has focused either on a small number of related languages (e.g., [5]) or on surveys across very distantly related languages (e.g., [3]). These are useful for producing highly detailed case-studies of well-understood systems or for evaluating the outer bounds of variation across languages in general. However, insights into historical dynamics at a statistically significant level are most readily obtained through phylogenetic analysis of large sets of related languages. Here, we fill a gap in the FL research by presenting the first phylogenetic study of FL and thereby produce the first results for historical dynamics that are supported at a statistical level across a large language family.

Research on FL has focused particularly on phonological contrasts whose FL is very low, for reasons related to a prominent conjecture in historical linguistics [1] (see Section 1.2). However, contrasts in human languages have a range of different functional loads, from very low to very high, and important questions include why this range exists and how FL values evolve over time. In terms of the evolutionary question, we can ask: (1) What kinds of changes to FL values appear to occur? (2) How do those changes pattern more broadly, for instance, does their distribution follow some well-described stochastic process? (3) What might the possible causes of change be that give rise to the distributions we see?

Answering these questions will ultimately require fitting together the pieces of a large puzzle. In this study, we contribute some central pieces. We produce some striking findings regarding the *phylogenetic signal* of FL. A phylogenetic signal is a measure of how close the match is—at the level of a whole language family—between (1) actual FL values and (2) what would be expected if FL was inherited from ancestor languages to their descendants while undergoing fluctuations according to a stochastic process termed Brownian motion (more on this in Section 2.2).

Naturally, we would not suppose that the real evolution of FL is so simplistic, rather the questions are: how close to such a scenario does the evolution of FL appear to come, and how does this compare to the evolution of other properties of language? Answering these questions provides insights into the kinds of explanations for FL evolution that could be consistent with the data. We also examine the correlation, or trade-off relationship, that can exist between the FL of two different contrasts in a linguistic system. Understanding how multiple, individual changes to FL are linked (or not) provides new insights into the historical dynamics of contrastiveness in linguistic systems.

### 1.2. Functional Load and Sound Change

Languages undergo mutational changes, known as sound changes, in which sounds may change from one phonemic category to another [13]. The term *unconditioned merger* refers to sound changes that cause two or more previously distinct phonemic categories to become conflated into one. *Conditioned mergers* are when conflation affects the phonemes only in certain contexts. FL has attracted attention as a possible explanatory factor in the incidence of mergers; it has long been conjectured [1] that contrasts with low FL are more prone to merge than contrasts with high FL, and recent results support that conjecture [5,14,15,16]. Debate is ongoing over which operationalizations of FL provide the greatest predictive power [5] and what kinds of mergers FL predicts [17]. Whether it used entropy-based definitions of FL or not, most research to date has focused on domains D that are words.

When a contrast undergoes an unconditional merger, its FL falls to zero. The fact that contrasts with low FL values are more likely to fall to zero than contrasts with higher FL values, is an observation that can be described in several ways, and each way of describing it can have different implications for what we believe is in need of explanation. For instance, taken by itself, it is consistent with a description in which (1) only falls to *zero* FL show a tendency for small changes in FL to be more likely than larger changes; (2) all *falls* in FL, whether the fall is to zero or otherwise, show the tendency; or (3) all *changes* to FL, whether they are falls or rises, show the tendency.

Since prior research has focused its attention on falls to zero, it has not yet been established whether or not current observations will generalize to support strictly case (1), the slightly broader case (2), or the general case (3). The study we present here will consider evidence for case (3). If it turns out that case (3) is supported, then this may recast how we think about recent results in sound change, since the phenomenon requiring explanation will not be only mergers but all changes in FL.

### 1.3. Contrastiveness in Pama–Nyungan VC Strings

In this paper, we examine FL from a phylogenetic perspective, investigating how FL evolves over time. Our empirical focus is in the large, Pama–Nyungan (PN) language family of Australia. PN languages extend across 90% of the Australian mainland and the time depth of the family is estimated at around 5000–6000 years before present [18,19,20].

In many PN languages, an inverse correlation has been observed between phonemic vowel length and the phonetic duration of a following consonant [21,22,23,24,25]. This is particularly so for vowels in the first syllable of words, referred to as tonic vowels. Here, we focus on tonic vowels and single, intervocalic consonants that follow them. Post-tonic single consonants that follow phonemically short vowels and that have phonetically longer durations in some languages also exhibit additional phonetic properties associated with long duration, such as more complete closure and passive devoicing of stops as well as pre-stopping of nasals and laterals.

Conversely, post-tonic single consonants that follow phonemically long vowels and that have phonetically shorter durations may exhibit more voicing and lenition of stops, and the absence of pre-stopping in laterals and nasals [21,22,26,27,28]. Examples of allophonic conditioning of this kind are cited in Table 1.

A general fact about sound change is that when two phonemes merge, it is possible for phonetic correlates of the original contrast, which are manifested in other segments, to remain in place and become contrastive. This has occurred in multiple branches of PN, as phonemic vowel length is lost while its erstwhile phonetic correlates on the following consonant remain and become distinctive. Examples are cited in Table 2.

In the cases cited in Table 2, the complete merger of the length contrast in the tonic vowel is associated with an increase in contrastiveness in following consonants. In such cases, the FL of the length contrast in tonic vowels falls (to zero) while the FL of manner of articulation contrasts (including voicing and fortition) in the following consonant rises. Consequently, there is a trade-off relationship between $FL_{V}$, the FL of tonic vowel length, and $FL_{C}$, the FL of post-tonic consonantal manner of articulation.

This trade-off is of a very specific kind, in which the complete merger of all short/long vowel pairs reduces $FL_{V}$ to zero. We will refer to this as a *trade-off with contrast collapse*. Other trade-offs are possible, however. If a length contrast is lost only in certain vowels, and/or only in certain contexts, then $FL_{V}$ would fall (though not to zero), and in such cases, $FL_{C}$ could be expected to rise if the consonants become more contrastive when they follow the vowels that do merge. This scenario would give rise to a second kind of trade-off. We will refer to this second kind as a *trade-off with contrast maintenance*.

The two studies that we present below examine the evolution of the FL of tonic vowel length contrasts and of contrasts in the following consonant in PN.

Study 1 establishes that these FL variables contain significant phylogenetic signals. One implication of this is that our data are consistent with an evolutionary process in which smaller changes in FL—both falls and rises—have been more common than larger changes. More detail is given in Section 2.2 below.

Study 2 examines FL trade-offs with contrast maintenance. We test the hypothesis that $FL_{V}$ and $FL_{C}$ are negatively correlated in PN. $FL_{C}$ is defined in terms of the manner of articulation of post-tonic consonants, and we expect $FL_{C}$ to correlate negatively with $FL_{V}$ for the phonetic and historical reasons introduced above. We also examine $FL_{P}$, the FL of place of articulation of post-tonic consonants. As research on PN languages has identified no particular association between vowel length and consonant place, our hypothesis is that $FL_{P}$ will show no significant correlation with $FL_{V}$.

## 2. Materials and Methods

### 2.1. Functional Load Data in Pama–Nyungan

We estimated the FL for vowel length ($FL_{V}$), consonant manner ($FL_{C}$), and consonant place ($FL_{P}$) in domains comprised of a tonic vowel followed by a single, intervocalic consonant in a set of 90 Pama–Nyungan languages listed in Appendix A. To calculate the FL, we used both the unnormalized formula in (2) and the normalized formula in (3). In cases where we intended to be specific, we refer to the unnormalized values as $FL_{V}^{u}$, $FL_{C}^{u}$, $FL_{P}^{u}$ and the normalized values as $FL_{V}^{n}$, $FL_{C}^{n}$, $FL_{P}^{n}$. In most cases though, when the point under discussion applies equally to both, we write $FL_{V}$, $FL_{C}$, $FL_{P}$.

At the current stage of documentation of global linguistic diversity, every major language family contains many low-resource languages for which data are scarce [41]. As a consequence, studies of FL, such as ours, which aim for a coverage that spans whole families, will face limits on the data available. This is true in the case of Pama–Nyungan. For most languages in our dataset, the available corpora are lexical lists. As we will see in Section 3, this does not prevent clear results from emerging, and we return to discuss the reasons why in Section 4.1.

**Figure 1:**
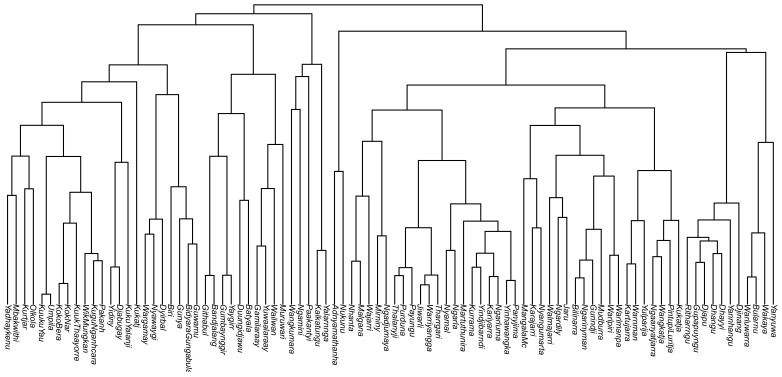
Pama–Nyungan tree containing the 90 languages used in this study, inferred from lexical cognacy judgements. Displayed here is a single, *maximum clade credibility* tree, i.e., the one tree within the 1000-tree sample that most adequately represents the most frequently recurring subgroups in all of the trees of the sample.

Correspondingly, our FL estimates were based on lexical datasets from which we extracted instances of the domain of interest. The lexicons contained between 208 and 3215 domain instances (mean 774 and median 605). The 90 languages studied were selected by taking the 112 PN lexicons studied in [42] and keeping only those that (1) have some degree of tonic vowel length contrast (since we want to study changes in FL that do not involve complete mergers) and (2) that have greater than 200 domain instances. A representative tree of these 90 languages is shown in Figure 1.

When classifying vowels as short or long, we regarded sequences of two adjacent short vowels as one long vowel, and sequences of /uwu/ and /iji/ as long vowels, since a tradition followed in some Australianist analysis is to represent long high vowels [u:] and [i:] as phonemic vowel-glide-vowel sequences (e.g., ([43], p. 24), ([44], p. 91)).

Phonemically long or geminate consonants, and phonemically pre-stopped sonorants, have been analysed as both mono- and bi-segmental units in the Australianist literature [45]. Here, we were guided by the kinds of historical developments that we wish to study, and we classed them as single segments.

The FL data obtained for the 90 PN languages are reported in Appendix A.

### 2.2. Phylogenetic Analysis

As languages are related to one another, it is not statistically valid to treat cross-linguistic observations as independent [46]. Quantitative phylogenetic methods [47] take genealogical relatedness into account in a principled and statistically sound manner. Our two studies use phylogenetic techniques in order to make valid inferences from the cross-linguistic FL data in PN.

**Study 1** assesses the degree of phylogenetic signal in $FL_{V}$, $FL_{C}$, and $FL_{P}$. The phylogenetic signal is a measure that compares the variation in an observed variable against its expected variation if it evolved along a phylogenetic tree, *t*, according to a Brownian motion process. In Brownian motion, the value of a variable is in constant flux. Positive and negative changes are equally likely at all times, and small changes are more likely than large ones. A phylogenetic signal will be stronger when the variable in question actually did evolve along the tree, and less strong if it was influenced by lateral transfer as in borrowing, especially borrowing among languages that are only distantly genealogically related.

The phylogenetic signal will be stronger when the variable evolved along the specific tree *t*, to which the data are being compared and not some other tree, $t^{\prime }$. Furthermore, it will be stronger when evolution was similar to Brownian motion, so that the value of the variable had equal probabilities of shifting up or down at any point, as opposed to (for example) being constrained within some tight range, so that at extreme outer values, there was a greater chance for the variable to evolve back towards the central value than further outwards. For a more technical description of phylogenetic signals written for a linguistic readership, see [42,46].

Here, we measure the phylogenetic signal, using the *picante* package in R [48], according to a standard two-step procedure described in Blomberg et al. [49]. First, the variation in the data is compared to a randomized baseline in which the shape of a previously determined tree *t* plays no role in structuring the data. The null hypothesis is that, relative to the structure implied by the tree, the data are simply random; the alternative hypothesis suggests patterns like the tree.

For example, a phylogenetic signal is considered to be evident at a p=0.05 level if the variation in the real data matches the tree better than 95% of the randomized datasets. Next, if the data has been confirmed as significantly differing from randomness, then we use the statistic, Blomberg’s *K* to measure the strength of phylogenetic signal. (For mathematical details of the calculation of *K*, see [42,49].) Blomberg’s *K* takes a value of 1 if the variation in the data accords perfectly with the tree *t*, and a minimum value of 0 if the data are perfectly randomly distributed relative to *t*. Values in excess of 1 are possible if FL data values are highly clumped within subgroups of the family.

When calculating a phylogenetic signal, reference must be made to a tree, *t*. In our case, the aim is to compare FL data to the PN family tree. However in linguistics, there is uncertainty regarding the details of this tree. Uncertainty about the details of trees is common in phylogenetic research and is termed *phylogenetic uncertainty*. Here, we employ a standard approach to account for phylogenetic uncertainty, by measuring the phylogenetic signal with respect to not one tree *t* but a sample of 1000 highly-likely family trees $t_{1},t_{2},\ldots ,t_{1000}$. This generates 1000 tests against randomness, followed by 1000 estimates of *K*, which provide a distribution describing its likely value. Our tree sample $t_{1},t_{2},\ldots ,t_{1000}$ comprises 1000 dated phylogenetic trees from the posterior distribution inferred by Bowern [50] and described further in Macklin-Cordes et al. [42]. The trees were inferred from cognate data from which known borrowings were excluded [19,20].

**Study 2** examined the phylogenetic Pearson’s correlation [51] between $FL_{V}$ and $FL_{C}$ and between $FL_{V}$ and $FL_{P}$. This test is conceptually parallel to a regular Pearson’s correlation; however, it also takes into account the specific kinds of non-independence caused by genealogical relationships between languages. As a consequence, the results reflect correlations not only between the values of traits in individual modern languages but also between values characteristic of subgroups at all levels in the tree. As such, the results for a pair of variables can inform us about the strength and direction of linked relationships that characterize the language family as a whole, through its history. When we calculate the statistics, as with our estimate of the phylogenetic signal, we take phylogenetic uncertainty into account by performing the correlation test in reference to the sample of 1000 highly-likely trees.

Most statistical tests require assumptions to be made about the data. The test we use in study 2 assumes that $FL_{V}$, $FL_{C}$, and $FL_{p}$ evolve along a phylogeny following Brownian motion. Given the results that we obtained in Study 1 for Blomberg’s *K* (see Section 3), the assumption is well motivated. We used the phytools R package [52] to estimate the covariance matrix of the Brownian motion on each tree in the sample, giving a sample from the posterior distribution of Pearson’s *r* correlation. The *p*-values reported were computed using the posterior mean estimates and correspond to testing the null hypothesis that the correlation is zero against the alternate hypothesis that the correlation is non-zero.

## 3. Results

**Study 1** The statistical significance of the presence of phylogenetic signal was measured to three digits of accuracy, and for all FL variables and all trees, the highest *p*-value was p=0.001, indicating that a phylogenetic signal was significantly present. The strength of the phylogenetic signal as measured by Blomberg’s *K* was very close to 1 for $FL_{V}$, $FL_{C}$, and $FL_{P}$, as shown in Table 3, both for the normalized and unnormalized versions of the FL measure.

To place these *K* values in context, Macklin-Cordes et al. [42] examined the lexical Markov chain transition probabilities of biphones (two-segment sequences) in PN and found mean *K* values of 0.54 or mean *K* of 0.59 when segments were binned into groups by place or manner of articulation. Macklin-Cordes and Round [46] examined the relative frequencies of dental versus palatal consonants in word initial and intervocalic positions in PN and found mean *K* values from 0.78 to 1.32 word-initially and from 0.34 to 0.70 intervocalically.

Dockum [53] examined phoneme frequencies and biphone Markov chain transition probabilities in languages of the Tai family and found mean *K* values of 0.71 and 0.68, respectively. Further afield, Blomberg et al. [49] examined 121 biological traits of a wide variety of plant and animal organisms, finding mean *K* values of 0.35 for behavioural traits, 0.54 for physiology, and 0.83 for traits related to body size. Taken in this context, our results suggest that the evolution of FL is very well described by Brownian motion process along the PN tree.

**Study 2** One consequence of the high levels of phylogenetic signal found in FL, is that statistical analysis, such as the measurement of correlations should be carried out using phylogenetic comparative methods [46]. Phylogenetic Pearson’s correlation (Table 4) was significant and negative between $FL_{V}$ and $FL_{C}$ but did not reach significance between $FL_{V}$ and $FL_{P}$, in accordance with our hypotheses. This was true for both for the normalized and unnormalized versions of FL.

## 4. Discussion

The studies in this paper have examined language diachrony at a statistical level. In doing so, we contribute to a more precise, quantitative characterization of diachronic typology. Specifically, we studied the historical dynamics of FL, which is a quantitative characterization of the contribution of specific contrasts to distinctiveness in the lexicon.

We established that the FL of certain variables evolves according to non-independent stochastic processes: they were found to change in a linked, statistically correlated fashion across almost a hundred languages and thousands of years of history. Moreover, we demonstrated that FL exhibited interesting historical dynamics that are deserving of further investigation not only at values close to zero, which have been the focus of prior work but at higher values as well.

Recent research has confirmed a long-standing conjecture that contrasts with low FL are particularly prone to merger [5,14,15,16], which is to say that FL is more likely to fall to zero from a lower value than from a higher one. Efforts at explaining this phenomenon have focused on homophony avoidance, an account that is specific to FL falling to zero and doing so in the domain of whole words [5,17]. However, our findings suggest that there may be nothing special about FL falls as opposed to rises, and changes to zero as opposed to other values. In addition, the word is not the only domain in which causally interesting effects of FL may be active.

Consequently, although it was not our primary focus here, our findings suggest that recent efforts may be focusing on too narrow a research question and consequently entertaining a set of explanatory accounts that will generalize only poorly to other, related phenomena. Future research will benefit from broadening its scope beyond the recent, more narrow focus on FL, which falls to zero in whole words.

We now take up three additional topics for expansion and emphasis.

### 4.1. High Degree of Phylogenetic Signal in FL

Phylogenetic signals have recently been shown to be present in phonotactic biphone frequencies, phoneme frequencies, and contextual ratios of places of articulation [42,46,53,54,55]. These studies reveal that the frequencies of phonological *structures* pattern with genealogy. Here, we find a high level of phylogenetic signals also in FL—that is, in the contrastive *function* that phonological structures serve. Interestingly, we find that the phylogenetic signal in the FL measures examined here was very close to 1 and closer than the values found in studies of phonological structures. Two questions can be posed in response: why did we find a strong phylogenetic signal in FL, and why is it even stronger than in structural traits? Any answer at this stage of research is necessarily speculative; however, the observations we offer here may point to useful lines of future inquiry.

Why did we find a strong phylogenetic signal in FL in the data that we used, bearing in mind that our data (1) are sourced from lexical lists of word types, not tokens; (2) are sourced from lists that are short, mostly numbering in the hundreds of items, not thousands or tens of thousands; and (3) examine FL in domains comprised of a tonic vowel and following consonant, not whole words. One prior expectation might have been that since the datasets are so small and since they do not examine the full words that are the focus of much recent research, they would be awash in statistical noise and exhibit little patterning of significance. Evidently, this is not the case, however, and we believe there may be reasons why not.

Much research on FL has focused on the hypothesis that FL exerts an influence on sound change through a mechanism of homophony avoidance [1,5,15,17]. Since homophony is a relationship that holds between *words*, the effect of such a mechanism may be to promote FL within the domain of whole words. Furthermore, as a consequence of that mechanism, sound changes would be less likely to occur, the more they altered the FL within words. However, whether this hypothesis is correct or not, other mechanisms should not be ruled out.

For instance, during speech processing, words become activated cognitively well before the listener hears the entire word [56]. Consequently, any sound change that caused a loss of a contrastiveness early in a word could potentially impair the ease of processing, even if it did not result in homophony. Accordingly, if we are prepared to entertain the existence of homophony avoidance as a causal factor in sound change, it is not unreasonable to entertain the existence of an avoidance of loss of contrastiveness early in the word as an additional factor in sound change (for supporting evidence, see [57,58]).

By this line of reasoning, since our data focuses on the first vowel and following consonant of PN words, i.e., contrastiveness early in the word, it is not altogether surprising that our results were significant despite the fact that we did not examine whole words. (PN roots are typically disyllabic and affixation is suffixal [59]; the initial consonant position, before the tonic vowel, permits only a subset of the contrastive consonants found elsewhere [45,60], which potentially increases the importance placed on maintaining the subsequent VC contrasts.)

Our data come from short word lists, which might be expected to supply FL values that are, at best, a noisy approximation of the more precise FL values obtainable from larger lists or from token-based corpus data [61,62]. However, the words that appear in short wordlists are heavily skewed towards the most frequent words of a language, and these are the words that listeners would process most often and would have learned the earliest during acquisition. If we grant that words of higher frequency and earlier acquisition are likely to play an especially significant role in the mechanisms behind sound change, then it follows that even short wordlists will plausibly contain rich evidence of the contrastiveness that matters most.

Our results also align with the findings of [61] in that, above a minimum threshold for wordlist length, even lists of only a few hundred words contained phonemic distributions that conformed closely to the full lexicon, when randomly sampled from a larger lexicon containing thousands of items. Thus, it is not as surprising as it might first seem that we obtained clear results and a strong phylogenetic signal from the limited data we had available.

Our second question was, why does the phylogenetic signal appear higher in FL than in structural traits of phonology, such as phonotactics? To answer this, we return to the two causes of stronger/weaker phylogenetic signals described in Section 2.2.

First, a phylogenetic signal is stronger when computed relative to the truest tree and lower otherwise. However, the trees we used here for PN while studying FL are the same as those used by Macklin-Cordes et al. [42] for phonotactics, and thus a difference in the trees used is unlikely to be the cause of the differences in phylogenetic signal.

Second, a phylogenetic signal is stronger if the change process has the properties of Brownian motion. In Brownian motion, small changes are more likely than large changes, and positive and negative changes are equally likely. We take these aspects in turn.

Both FL and structural traits—as with phonotactic frequencies—change as the lexicon changes. Any lexicon is constantly affected in small ways by neologisms and the obsolescence of words. Additionally, they may be affected by borrowing, which can occur at various rates, and by sound changes, which can occur in highly specific contexts or more sweeping ones. This mixture of factors supports an expectation that small changes will be frequent and larger changes less so, and it is not obvious that there would be significant differences in this regard between FL or traits, such as phonotactic frequencies.

It now remains to consider whether positive and negative changes in values are equally likely. For FL, positive/negative changes in values entail that a contrast becomes more/less central in supporting the distinctiveness of strings in the lexicon. There is a lower bound at zero; however, in our study, we did not include that lower bound. Aside from that lower bound, we are not aware of constraints that would make the likelihood of positive or negative changes uneven at any point, and consequently the stochastic process that describes changes in FL could genuinely be quite close to Brownian motion. For structural aspects of phonology, however, the situation is different.

Structural features are subject to constraints: there are less likely and more likely structures, both in universal and in lineage-specific terms [45,63]. Consequently, for instance, the frequency of a highly marked structure should be more likely to decrease than to increase. This kind of inequality in the likelihood of positive and negative changes to values—irrespective of the actual sources, such as production, perception, and cognition—entails a departure from Brownian motion, which ought to weaken the phylogenetic signal. This, we suggest, may be why structural traits appear to have a lower phylogenetic signal compared with FL. If this line of reasoning is correct, we would expect similar results to emerge from studies of other language families beyond PN.

### 4.2. Transphonologization and the Flow of Contrastiveness

Transphonologization [64] (cf. rephonologization [65] and cheshirization [66]) is a term given to sound changes in which a contrastive function is preserved; however, the locus of the contrast—the segments or features that instantiate it—changes. Here, we studied a closely related phenomenon in which contrasts do not necessarily disappear or emerge in their entirety, but the relative contrastive workload of them (their FL) does shift from one to another. One way to view this phenomenon is in terms of a diachronic *flow* of FL from one contrast to another (cf. [67]). The fact that we are able to quantitatively detect the presence of this flow of contrastiveness through a language family as large and old as PN suggests the potential of new avenues for investigating the dynamic flow of contrastiveness through phonological systems as they evolve over time.

One question that arises is whether our findings in PN might reflect some strong preference in language for the conservation of contrastiveness in which case, the flow of FL from one contrast to another might be regarded as an automatic consequence of one contrast undergoing a significant decrease in FL. Although our results alone cannot answer this question, we doubt that such a principle exists in any strong form. Certainly, in many mergers, the overall contrastive capacity of a language is simply reduced, as the FL of one contrast falls but no other FL rises to balance it.

In the case of PN tonic vowels and post-tonic consonants, we suggest that the cause of recurrent historical flow of FL lies in particular phonetic factors that are common across PN languages: a synchronic correlation between phonetic tonic vowel duration and phonetic post-tonic consonant manner, even in systems in which only the vowel-durational aspect is tied to a synchronic phonemic contrast; when the phonetic vowel-durational differences are neutralized diachronically, causing the phonemic vowel length contrasts to collapse, the phonetic manner differences—which still correlate with the same lexical distinctions that vowel length had signalled—become phonemic. On this view, it is the phonetics of the vowel–consonant strings that furnish the conditions for a natural flow of FL from vowel to consonant.

### 4.3. On Sapir’s ‘Drift’: The Non-Accidental, Parallel Evolution of Related Languages

It is a century now since the appearance in print of Edward Sapir’s hypothesis that languages undergo parallel grammatical evolution for several centuries after they split [68]. Providing anything more than anecdotal evidence in support of Sapir’s hypothesis has long been difficult [69,70,71], and some apparent cases may be due to language contact [72,73]. Dunn et al. [74] used phylogenetic methods to examine patterns of word order evolution in different language families; however, the study did not produce an identifiable cause for those patterns.

Ideally, evidence in support of Sapir’s drift should not be anecdotal but rather be statistically significant across a language family; it should not be reducible to the effects of language contact, and it should be relatable to an identifiable cause. The current study meets these three criteria. It detects parallel changes in FL that are instantiated statistically across 90 languages within the PN family whose time depth is estimated at around 5000–6000 years [18,19,20]; thus, the evidence is not anecdotal.

The data pattern fits tightly with phylogeny, and thus is unlikely to be due to contact (cf. our note in the next paragraph). Furthermore, we identified a causal basis for this, in the common phonetics of PN tonic vowel–consonant sequences. Thus, we believe our results to be one of the fullest confirmations yet that Sapir’s conjecture was essentially correct: that under the right circumstances, linguistic systems can undergo parallel evolution after they split, not merely for centuries but for millennia.

A reviewer asks about the situation in which language contact closely mimics the pattern of phylogeny. If contact did pattern perfectly with phylogeny (such that languages only borrowed from their very closest relatives), then its effects would be indistinguishable. However, languages also borrow from geographic neighbours that are less closely related. It is hard to conceive of borrowing of lexical items whose impact on contrastiveness has the phylogenetic signal we find here in the absence of vertical inheritance. At the very least, the burden of proof is on the advocate of a language-contact account, given that the data pattern is in very close accord with expectations from vertical inheritance, and there is an accompanying explanation in terms of phonetics and sound change for why this should be so.

It has been suggested by Joseph [75] that drift in phonology may be due to a narrowing of the range of variation inherited from a proto-language. In the PN changes described here, however, the flow of FL from vowel length to consonant manner is not due to any narrowing of variation in FL in proto-PN (indeed it is not entirely clear what it should mean for FL to have a range of variation). Nor, when the PN developments are viewed in terms of phonological substance are they a matter merely of narrowing variation. Although contrasts in vowel length are lost, new variation is introduced in the inventory of contrastive consonant manners and into the set of relationships that can hold between the length of a tonic vowel and the manner of post-tonic consonants.

In reality, Joseph’s proposal would appear to reduce to a fact, well-recognised in evolutionary biology, that incomplete lineage sorting (i.e., the inheritance of variation from a proto-taxon into its descendant) can result in the appearance of convergent evolution [76]. However, this does not entail that all convergent evolution is due to incomplete lineage sorting (see also [77]).

Another important source can be the existence of dependencies within a system that are inherited along with its substance [78], which will favour certain outcomes over others in descendent systems as has been observed in protein evolution; for example, ref. [79]. In PN, certain phonetic dependencies between tonic vowel length and post-tonic consonant manners were inherited alongside the phonological substance itself. In the descendent systems, in the event that vowel length was lost, the inherited dependencies favoured the rise of new, contrastive consonant manners.

## 5. Conclusions

This paper joins a growing body of work regarding the application of computational phylogenetic methods to phonological data. It also represents the first phylogenetic study of FL.

We showed that there was a significant phylogenetic signal in FL, which has implications for a better understanding of the dynamics of sound change. Further, we showed that the FL of tonic vowels and post-tonic consonants were negatively correlated in PN and that this maps closely to a sample of highly probable PN family trees. We also introduce the idea of the *flow* of contrastiveness between subsystems of the phonology in different languages, which is connected to the concept of transphonologization, and we claim that this represents a concrete example of Sapir’s drift.

Setting our gaze beyond PN, while not all sound changes are associated with the phonetic conditions that promote the flow of FL, there are many that appear to be, and it will be valuable to apply the methods we introduced here to study them. In time, this may lead to a more general understanding of how FL can flow within phonological systems over long time horizons. Promising future applications of our approach include the investigation of other suspected diachronic trade-offs, such as the rise of phonemic tone and register (such as contrastive phonation) in Southeast Asia tied to losses of consonantal laryngeal distinctions [3,80,81,82].

## Figures and Tables

**Table 1:** Examples of allophony in post-tonic consonants conditioned by the phonemic length of the tonic vowel. Key: V_ after a phonemically short vowel, VV_ after a phonemically long vowel. See references for additional details in the conditioning of allophony.

Language (Subgroup)	Consonants	V_	VV_
Djambarrpuyngu (Yolngu) [25]	Consonants	longer	shorter
Wik (Middle Paman) [29]	Stops	tenser	laxer
Kugu Nganhcara (Middle Paman) [30]	Voiced stops	stop	fricative
Nukunu (Thura–Yura) [31]	Nasals, Laterals	prestopped	plain
Yadhaykenu (Nothern Paman) [32]	Laterals	plain	flapped

**Table 2:** Examples of post-tonic consonant contrasts created upon the merger of length distinctions in tonic vowels of Pama–Nyungan languages. Key: T short stop, TT long stop, D voiced stop, Z spirant, N nasal, NN long nasal, DN prestopped nasal, ND nasal+stop, L lateral, DL prestopped lateral, V_ after erstwhile short vowel, and VV_ after erstwhile long vowel. See the references for additional details and conditioning of the tabulated sound changes.

Language (Subgroup)	Original C	V_	VV_
Warumungu (Warunmungic) [33]	T	TT	T
Wik-Muminh (Middle Paman) [29]	T (non-apical)	T	D
Northern Paman subgroup [32,34,35]	T (non-apical)	T	Z
Lamalama, Umbuygamu (Lamalamic) [36]	/k/	/k/	/h/
Kugu Mumminh (Middle Paman) [33]	N	NN	N
Arandic subgroup [37,38]	N	DN	N
Walangama (Norman Paman) [39]	N	DN	N
Olgolo (Southwest Paman) [40]	N	DN	N
Lamalama (Lamalamic) [36]	N	ND	N
Rimanggudinhma (Lamalamic) [36]	N	D	N
Thura–Yura subgroup [31]	L	DL	L

**Table 3:** Phylogenetic signal in $FL_{V}$, $FL_{C}$, and $FL_{P}$, measured using Blomberg’s *K* and a sample of 1000 reference PN trees.

Functional Load Measure	Mean *K*	std.dev of *K*
unnormalized FL		
FL of tonic vowel length ($FL_{V}^{u}$)	0.972	0.036
FL of post-tonic consonant manner ($FL_{C}^{u}$)	0.956	0.038
FL of post-tonic consonant place ($FL_{P}^{u}$)	0.960	0.030
normalized FL		
FL of tonic vowel length ($FL_{V}^{n}$)	0.997	0.039
FL of post-tonic consonant manner ($FL_{C}^{n}$)	1.181	0.035
FL of post-tonic consonant place ($FL_{P}^{n}$)	1.010	0.033

**Table 4:** Phylogenetic Pearson’s correlation between $FL_{V}$, $FL_{C}$, and $FL_{P}$.

Functional Load Measures	*r*	95% Interval	*p*
unnormalized FL			
$FL_{V}^{u}$ versus $FL_{C}^{u}$	−0.28	[−0.46−0.08]	0.006
$FL_{V}^{u}$ versus $FL_{P}^{u}$	0.03	[−0.180.23]	0.78
normalized FL			
$FL_{V}^{n}$ versus $FL_{C}^{n}$	−0.50	[−0.64−0.33]	$5\times 10^{-7}$
$FL_{V}^{n}$ versus $FL_{P}^{n}$	−0.19	[−0.380.02]	0.08

## Data Availability

FL data are supplied in Appendix A. PN trees were published with [42] and are available at https://zenodo.org/record/3988775 (accessed on 15 December 2021).

## Abbreviations

The following abbreviations are used in this manuscript:

FL	Functional load
PN	Pama–Nyungan

## Appendix A. Functional Load Data

**Table A1:** Functional load estimates and the number of observations on which they are based in Pama–Nyungan languages A–M.

Language	${FL}_{V}^{u}$	${FL}_{C}^{u}$	${FL}_{P}^{u}$	${FL}_{V}^{n}$	${FL}_{C}^{n}$	${FL}_{P}^{n}$	N
Adnyamathanha	0.00728	2.29877	1.69328	0.00119	0.37569	0.27673	1424
Anguthimri	0.15786	0.89040	0.94600	0.02788	0.15725	0.16707	255
Bakanh	0.48216	0.75675	1.70156	0.08106	0.12723	0.28607	379
Bidyara	0.02048	1.04399	1.43908	0.00418	0.21327	0.29397	466
Bilinarra	0.02401	1.60119	1.38537	0.00454	0.30258	0.26180	1071
Biri	0.01129	1.04712	1.30791	0.00225	0.20869	0.26067	387
Bularnu	0.01029	1.81420	1.53169	0.00182	0.32136	0.27131	514
Butchulla	0.15259	1.20493	0.83980	0.03047	0.24058	0.16768	303
Dhangu	0.53689	1.30565	1.20428	0.09303	0.22623	0.20866	222
Dhay’yi	0.61997	1.33455	1.35632	0.10625	0.22872	0.23245	636
Djabugay	0.05108	1.26909	1.16098	0.01032	0.25636	0.23452	641
Djapu	0.55242	1.42580	1.45573	0.09347	0.24125	0.24632	563
Djinang	0.00835	1.75735	1.27597	0.00153	0.32242	0.23410	1459
Duungidjawu	0.35925	0.93156	0.91288	0.06575	0.17050	0.16709	340
Dyirbal	0.01899	1.20677	1.22623	0.00394	0.25013	0.25416	339
Gamilaraay	0.47639	1.09200	0.66671	0.09232	0.21161	0.12920	619
Gidabal	0.38315	1.20855	0.84965	0.07695	0.24271	0.17063	863
Gumbaynggir	0.68190	1.14161	0.56069	0.12949	0.21678	0.10647	260
Gunya	0.08592	1.67525	1.87928	0.01492	0.29087	0.32629	421
Gupapuyngu	0.70325	1.65965	1.38970	0.11275	0.26610	0.22281	1426
Gurindji	0.14136	1.68390	1.40002	0.02591	0.30864	0.25661	2783
Guwamu	0.15154	0.97184	1.28326	0.03000	0.19238	0.25402	284
Jaru	0.14610	1.65095	1.23854	0.02741	0.30974	0.23237	1459
Jiwarli	0.10655	1.49967	1.54472	0.01963	0.27631	0.28460	747
Kalkatungu	0.17358	1.45094	1.66749	0.03086	0.25799	0.29650	937
Karajarri	0.01130	1.58514	1.37181	0.00220	0.30850	0.26698	1217
Kariyarra	0.00794	1.51189	1.34208	0.00153	0.29066	0.25802	252
Kartujarra	0.10259	1.59830	1.62878	0.01872	0.29170	0.29726	526
Kok Nar	0.02440	1.02459	1.03786	0.00447	0.18781	0.19024	213
Koko Bera	0.00760	1.12514	1.14449	0.00139	0.20631	0.20985	428
Kugu Nganhcara	0.23071	1.20115	1.52446	0.03814	0.19859	0.25204	359
Kukatj	0.49247	1.37132	1.26212	0.08151	0.22696	0.20888	422
Kukatja	0.17702	1.69195	1.55646	0.03164	0.30243	0.27821	2339
Kuku Yalanji	0.00555	1.20557	1.06402	0.00113	0.24462	0.21590	1070
Kurrama	0.15740	1.47509	1.33256	0.02918	0.27348	0.24705	495
Kurtjar	0.35694	1.09790	0.90245	0.05715	0.17579	0.14450	405
Kuugu Ya’u	0.82180	0.96787	1.82687	0.14159	0.16676	0.31476	672
Malkana	0.09060	1.35974	1.51980	0.01719	0.25805	0.28843	208
Mangala	0.06827	1.58519	1.31169	0.01312	0.30471	0.25214	749
Martuthunira	0.12168	1.59684	1.41252	0.02230	0.29269	0.25891	633
Mirniny	0.05723	1.49353	1.64556	0.01071	0.27940	0.30784	259
Mudburra	0.12114	1.57266	1.36196	0.02255	0.29269	0.25347	509
Muruwari	0.29888	1.30293	1.23929	0.05555	0.24215	0.23032	873

**Table A2:** Functional load estimates and the number of observations on which they are based in Pama–Nyungan languages N–Z.

Language	${FL}_{V}^{u}$	${FL}_{C}^{u}$	${FL}_{P}^{u}$	${FL}_{V}^{n}$	${FL}_{C}^{n}$	${FL}_{P}^{n}$	N
Ngaanyatjarra	0.30847	1.65731	1.54372	0.05400	0.29015	0.27026	1125
Ngadjunmaya	0.27871	1.58111	1.52464	0.05123	0.29060	0.28022	512
Ngamini	0.00859	1.65769	1.62787	0.00167	0.32135	0.31556	453
Ngardily	0.04398	1.54071	1.41918	0.00838	0.29355	0.27040	274
Ngarinyman	0.04977	1.60920	1.37125	0.00949	0.30687	0.26150	870
Ngarla	0.05433	1.65036	1.52167	0.01026	0.31151	0.28721	940
Ngarluma	0.00949	1.54984	1.51068	0.00179	0.29179	0.28442	633
Nhanda	0.22128	1.61692	1.98274	0.03762	0.27491	0.33710	427
Nhangu	0.52706	1.62134	1.35121	0.08822	0.27137	0.22616	952
Nukunu	0.22996	1.88475	1.57220	0.03797	0.31122	0.25961	282
Nyamal	0.01096	1.66757	1.58458	0.00210	0.31958	0.30368	574
Nyangumarta	0.05591	1.63689	1.53035	0.01040	0.30451	0.28469	1002
Nyawaygi	0.44779	0.91693	0.84704	0.08627	0.17665	0.16318	339
Olkol	0.00913	1.89732	1.52545	0.00140	0.29102	0.23398	992
Panyjima	0.03319	1.52554	1.53691	0.00621	0.28545	0.28758	327
Payungu	0.12035	1.44293	1.48710	0.02226	0.26693	0.27510	514
Pintupi	0.25729	1.62037	1.49798	0.04605	0.29003	0.26812	3065
Purduna	0.18607	1.48411	1.64113	0.03364	0.26835	0.29675	540
Ritharrngu	0.52798	1.59391	1.28479	0.08893	0.26846	0.21640	841
Sth. Paakintyi	0.48182	1.31033	1.58642	0.08352	0.22713	0.27499	748
Thaayorre	0.39795	0.94581	1.29668	0.06750	0.16043	0.21995	873
Thalanyji	0.11295	1.41200	1.63985	0.02083	0.26037	0.30239	467
Tharrkari	0.10105	1.80780	1.46815	0.01760	0.31484	0.25569	371
Umpila	0.72633	0.89534	1.79211	0.12688	0.15640	0.31305	473
Waalubal	0.38811	1.21112	0.84641	0.07790	0.24309	0.16989	864
Walmajarri	0.07529	1.69469	1.53069	0.01387	0.31216	0.28195	2361
Wangkatja	0.24288	1.64652	1.54871	0.04329	0.29345	0.27602	1290
Wangkumara	0.03737	1.78415	1.49204	0.00662	0.31614	0.26438	480
Warlmanpa	0.03193	1.76883	1.54636	0.00582	0.32218	0.28166	603
Warlpiri	0.07788	1.71719	1.54913	0.01416	0.31215	0.28160	3215
Warluwarra	0.06211	1.62498	1.60851	0.01112	0.29084	0.28789	731
Warnman	0.03387	1.61920	1.55705	0.00628	0.30022	0.28870	607
Warrgamay	0.46606	1.16642	1.22445	0.08702	0.21779	0.22862	470
Warriyangga	0.04881	1.46336	1.55668	0.00925	0.27739	0.29508	273
Watjarri	0.09550	1.55502	1.58478	0.01742	0.28363	0.28906	787
Wayilwan	0.44092	1.11222	0.69925	0.08697	0.21937	0.13792	489
Western Wakaya	0.02551	1.47790	1.34683	0.00473	0.27429	0.24996	696
Wik Mungkan	0.57906	0.91075	1.73335	0.09237	0.14529	0.27651	1411
Yadhaykenu	0.11810	1.39036	1.47400	0.02185	0.25730	0.27278	385
Yalarnnga	0.01281	1.47869	1.59614	0.00239	0.27589	0.29780	397
Yanyuwa	0.02768	1.53369	1.47708	0.00521	0.28837	0.27773	1351
Yaygir	0.62734	1.49029	1.00410	0.11443	0.27184	0.18315	658
Yidiny	0.01376	1.24001	1.08403	0.00278	0.25063	0.21910	964
Yindjibarndi	0.12691	1.47608	1.21215	0.02391	0.27804	0.22833	492
Yinhawangka	0.08024	1.65358	1.67820	0.01442	0.29713	0.30155	773
Yulparija	0.07799	1.65715	1.58184	0.01426	0.30302	0.28925	1234
Yuwaalaraay	0.61024	1.18542	0.80690	0.11517	0.22373	0.15229	1109
